# Immediate fortification of human milk with a bovine milk-derived human milk fortifier in very low birth weight infants: a randomized clinical trial

**DOI:** 10.1038/s41372-024-01998-0

**Published:** 2024-05-09

**Authors:** Zanna Wynter, Jane Anne Gorham, Amy B. Thompson, Cynthia Mundy, Jennifer L. Waller, Brian K. Stansfield

**Affiliations:** 1 Department of Pediatrics, Augusta University, Augusta, GA USA; 2 Department of Family and Community Medicine, Augusta University, Augusta, GA USA

**Keywords:** Paediatrics, Outcomes research

## Abstract

**Objective:**

Double-blind randomized control trial of early addition of a bovine milk-derived human milk fortifier (HMF) in very low birth weight (VLBW) infants (NCT05228535).

**Methods:**

VLBW infants were randomized to receive bovine milk-derived HMF with first feedings or delayed fortification at 80 ml/kg/day. Anthropometrics were assessed weekly through 36 weeks postmenstrual age (PMA). Unadjusted and adjusted (race, gender, gestational age, and birth weight) differences between study arms were examined using two-sample *t*-test and ANCOVA, respectively.

**Results:**

Fifty-two VLBW infants (57% female, 60% Black) were enrolled. Baseline demographics did not differ between groups. Weight velocity at DOL 28 did not differ between study arms. Secondary outcomes including NPO occurrence, incidence of metabolic acidosis, NEC, retinopathy, or late-onset sepsis did not differ between groups.

**Conclusion:**

Immediate fortification of enteral feedings with a bovine milk-derived HMF appears safe and well-tolerated although no clear growth benefit could be established.

## Introduction

Mother’s own milk is considered the standard of care for very low birth weight infants (VLBW, <1500 g) and key stakeholders in infant nutrition recommend pasteurized donor human milk as the preferred alternative [1–3]. These recommendations are in line with clinical practice wherein the vast majority of neonatal intensive care units (NICU) use human milk as the primary diet for VLBW infants [4]. However, both mother’s own milk and donor human milk do not provide sufficient macro- and micronutrient content to support physiologic growth in VLBW infants [5–7]. In response to this clinical need, multi-nutrient human milk fortifiers (HMF) have been developed to supplement human milk and are widely used [8–10].

HMFs are sourced from bovine and human milk. Although HMFs are widely used, current practice holds that HMFs should be added to human milk once feedings are established, which is generally considered to occur once feeding volumes reach 60–100 ml/kg/day [11, 12]. However, the timing of human milk fortification is not well-supported. Recently, Salas et al. reported that addition of a human milk-derived HMF on feeding day 2 in preterm infants less than 28 weeks postmenstrual age (PMA) at birth resulted in greater length and weight gains without altering fat-free mass at 36 weeks PMA [13]. Importantly, the study failed to demonstrate a difference in the rate of necrotizing enterocolitis (NEC), spontaneous intestinal perforation (SIP), or death. These results mirror a previous open-label multi-center clinical trial and retrospective studies of early addition of a human milk-derived HMF to human milk [14, 15]. A similar open-label clinical trial compared early addition of an acidified bovine milk-derived HMF at a feeding volume of 20 ml/kg/day with delayed fortification at 100 ml/kg/day in VLBW infants with modest advantages in weight gain noted in the early fortification group [16]. However, early, unmasked addition of a powder bovine milk-derived HMF to human milk feeds failed to identify any growth benefits in preterm infants [17].

While these studies raise an important consideration that early addition of an HMF may provide some growth benefits in preterm infants, human milk-derived HMFs represent less than half of the market share [10] and the addition of a human milk-derived HMF to human milk is likely to be viewed as “low risk”. In contrast, bovine milk-derived HMFs are much more widely used, and limited evidence has been provided to guide the timing of their use. Here, we report growth and feeding-related outcomes from a double-blind randomized control trial to test the hypothesis that a bovine milk-derived HMF added to the first feeds would result in improved weight velocity with equivalent tolerability to delayed fortification at 80 ml/kg/day.

## Methods

This study was approved by the Institutional Review Board at Augusta University and registered as a clinical trial (NCT05228535). Interventions and study outcomes were performed in accordance with the Declaration of Helsinki. Study eligibility included 1) birth weight between 1000 and 1500 g, 2) admission to the NICU within 24 h of birth, and 3) maternal intent to supply breast milk and informed consent for the use of donor human milk. Infants were excluded from the study if 1) their birth weight was below the 3rd percentile for gestational age and sex on the Fenton Growth Curve, 2) enteral feedings were not initiated within 72 h of birth, or 3) they had major congenital anomalies that might impact feeding, growth, or survival. All preterm infants with a birth weight below 1500 g were screened for eligibility. Twins were allocated to the same study arm.

Parents of eligible preterm infants were approached for consent within 24 h after birth and prior to the initiation of enteral feeds. Upon study consent, infants were randomly assigned to one of two groups: 1) Intervention: bovine milk-derived HMF added to the first enteral feed or 2) Control: bovine milk-derived HMF added to feedings upon reaching 80 ml/kg/day. Infants were randomized in blocks of four using numbered, sealed opaque envelopes. Each envelope was opened sequentially by the Director of the Milk Laboratory at the Children’s Hospital of Georgia and allocation was communicated to two dedicated milk laboratory technicians who prepared all human milk for study participants but had no direct role in patient care. Human milk prepared for study infants was labeled “study” with no designation of calorie or nutrient content and was provided to nursing staff as per routine enteral nutrition schedule. All physicians, nurses, dieticians, and parents were blinded to study intervention.

All study infants were provided 20 ml/kg/day of mother’s own milk or donor human milk (Ni-Q HDM Plus™, 20 kcal/ounce, Wilsonville, OR) and advanced by 20 ml/kg/day every other day with a goal feeding volume of 140–150 ml/kg/day. Infants allocated to the intervention arm (designated “Early” fortification) received the bovine milk-derived HMF (Enfamil® Liquid Human Milk Fortifier Standard Protein, Mead Johnson Nutrition, Evansville, IN) beginning with the initial enteral feedings (5 mL HMF + 50 mL human milk, 22 kcal/ounce) according to the manufacturer’s instructions. Infants in the intervention arm were maintained on this recipe until feeding day 10 when they received human milk containing bovine milk-derived HMF (5 mL HMF + 25 mL human milk, 24 kcal/ounce) according to the manufacturer’s instructions. Infants in the control arm were provided human milk (20 ml/kg/day) without HMF and advanced by 20 ml/kg/day every other day until 80 ml/kg/day. After achieving an enteral feeding volume of 80 ml/kg/day for 24 h, infants in the control arm (designated “Late” fortification) were provided human milk with the bovine milk-derived HMF (5 mL HMF + 50 mL human milk, 22 kcal/ounce) and advanced to the higher caloric density (5 mL HMF + 25 mL human milk, 24 kcal/ounce) 48 h later. All study infants were provided NaCl (1.3 meq/kg/day), MgCO_3_ (6 mg/kg/day), Zinc (1 mg/kg/day), iron (6 mg/kg/day), vitamin D (800 IU/day) and total parenteral nutrition (TPN) as per usual care. Study infants were maintained on human milk containing HMF (24 kcal/ounce) until hospital discharge, if receiving mother’s own milk, or upon reaching 1800 g were transitioned to preterm infant formula (Enfamil® Premature, 24 kcal/ounce, Mead Johnson, Evansville, IN).

Anthropometric measurements were performed by the same 2 study members (ZW, JAG) for all study infants. Weight, length, and head circumference were measured within 24 h of birth and every 7 days (+/− 1 day) through 28 days of life and at 36 weeks PMA or at the time of hospital discharge, whichever occurred first. Mid-arm circumference was measured at 28 days of life. Weight was measured twice on a tared infant scale calibrated to the nearest 10 g. Discrepancies of greater than 10 g were repeated, and the average weight (g) was recorded. Head circumference was measured with a Seca® non-stretch, Teflon® measuring tape. The tape was applied firmly around the head above the supraorbital ridges, covering the most prominent part of the frontal bulge anteriorly, and over the part of the occiput that gives the maximum circumference. Discrepancies of greater than 0.5 cm were repeated, and the average head circumference (cm) was recorded. Length was measured on a length board with a stationary headpiece and adjustable footpiece. One examiner held the infant’s head with the Frankfurt plane in the vertical position and applied gentle traction to bring the top of the head into contact with the fixed headboard. The second examiner held the infant’s feet, toes pointing directly upward, and applied gentle traction to bring the movable footboard to rest firmly against the infant’s heels. Discrepancies of greater than 0.4 cm were repeated, and the average length (cm) was recorded. The right upper arm circumference was measured with a Seca® non-stretch, Teflon® measuring tape. Two measurements were obtained, and the average mid-arm circumference (cm) was recorded. Weight velocity was calculated using the following formula:

$$GV(g/kg/day)=\frac{1000\times ({W}_{n}-{W}_{birth})}{({D}_{n}-{D}_{birth})\times \frac{({W}_{n}+{W}_{birth})}{2}}$$

where

*W*_birth_ = weight at birth (g)

*W*_n_ = weight at day of measurement

*D*_birth_ = date of birth

*D*_n_ = date for measurement corresponding to DOL 28 or date of discharge/ 36 weeks PMA

Length and head circumference velocity (cm/week) were also calculated. Demographic data for all study participants included race, gender, APGAR scores at 1 and 5 min of life, total human milk intake and volume of mother’s own milk and/or donor human milk intake, days of TPN, number of missed enteral feeds, number of stools per day, emesis volume and events per day, and feeding-related days of *nil per os* (NPO). Incidence and stage of NEC [18], oxygen requirement at 36 weeks PMA, any retinopathy, incidence of metabolic acidosis (HCO_3_ < 18 mEq/L), and culture-positive sepsis were recorded.

### Sample size and statistical analysis

The primary outcome for the clinical trial was weight velocity (g/kg/day) at 28 days of life. A sample size calculation (alpha = 0.05, beta = 20%) based on a between-group difference in weight velocity of 3 g/kg/day (pilot sample of ten eligible infants, 10.6 ± 3.64 g/kg/day) determined that 50 infants (*n* = 25/group) would be necessary to determine superiority. Secondary outcomes included incidence of metabolic acidosis, feeding intolerance, incidence of NEC, oxygen requirement at 36 weeks, any retinopathy, culture-positive sepsis, and adiposity based on mid-arm circumference. All statistical analysis was performed using SAS 9.4 and statistical significance was assessed using an alpha level of 0.05. Descriptive statistics were calculated within group (Early or Late). Unadjusted differences between groups on weight velocity on day 28 and weight velocity at 36 weeks were examined using a two-sample *t*-test. To adjust for race, gender, gestational age at birth, and birth weight, ANCOVA was used to examine differences in weight velocity on day 28 and weight velocity at 36 weeks PMA between groups.

## Results

**Fig. 1:**
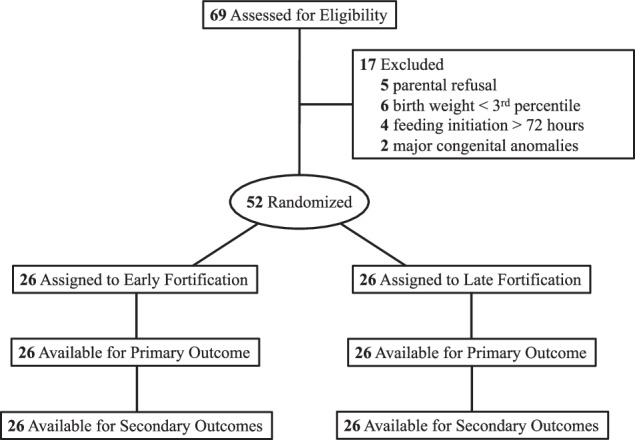
Study eligibility, screening, enrollment, and completion.

A total of 69 infants were assessed for eligibility between April 1, 2022, and December 31, 2023, and 52 infants underwent randomization with 26 participants allocated to each of the “Early” and “Late” fortification arms (Fig. 1). Baseline demographics for study participants are provided in Table 1. The mean birth weight and gestational age at birth for the entire study cohort (57% female, 60% Black) was 1285 ± 148 g and 214 ± 11 days, respectively. Birth weight was modestly higher (*p* = 0.09) as were length and head circumference (*p* = 0.04) in the Early group when compared with the Late group, but no statistically significant differences between groups were identified. All 52 participants reached the primary outcome of weight velocity at DOL 28 with no statistically significant difference between groups were identified (*t*_(50)_ = −0.08, *p* = 0.9348). Similarly, length and head circumference velocity did not differ between groups at DOL 28. Controlling for race, gender, gestational age at birth, and birth weight, weight velocity at DOL 28 did not differ between groups (Table 2).

**Table 1:** Descriptive statistics by fortifier group.

	Early (*n* = 26)	Late (*n* = 26)	*p*-value
EGA at birth (d)	214 ± 11	214 ± 12	0.81
EGA at discharge (d)	263 ± 17	264 ± 13	0.78
Female	11 (42.3)	19 (73.1)	0.05
Race			
Black	13 (50.0)	18 (69.2)	0.35
White	10 (38.5)	7 (26.9)	
Other	3 (11.5)	1 (3.9)	
Multiple birth	5 (19.2)	7 (26.9)	0.51
Maternal Age (yrs)	27.1 ± 6.4	26.9 ± 5.9	0.87
APGAR at 5 min^a^	7.0 (6.0–8.0)	7.0 (6.0–8.0)	0.87
Birth measurements			
Weight (g)	1320 ± 144	1250 ± 147	0.09
Weight Z-score	−0.43 ± 0.68	−0.48 ± 0.97	0.84
Length (cm)	38.3 ± 1.3	37.4 ± 1.7	0.04
Length Z-score	−0.60 ± 0.74	−0.82 ± 0.99	0.36
Head circumference (cm)	27.0 ± 1.2	26.3 ± 1.3	0.04
Head circumference Z-score	−0.62 ± 0.85	−0.89 ± 0.75	0.23
Growth velocities			
Weight velocity at 28 d (g/kg/day)	13.3 ± 2.8	13.4 ± 2.7	0.93
Weight velocity at 36 weeks (g/kg/day)	13.5 ± 2.6	13.9 ± 2.7	0.58
Length velocity at 28 d (cm/week)	1.0 ± 0.2	1.0 ± 0.2	0.93
Length velocity at 36 weeks (cm/week)	1.1 ± 0.2	1.1 ± 0.2	0.76
Head circumference velocity at 28 d (cm/week)	0.8 ± 0.2	0.8 ± 0.2	0.52
Head circumference velocity at 36 weeks (cm/week)	0.8 ± 0.2	0.9 ± 0.2	0.66
Mid-arm circumference			
28 days (cm)	8.0 ± 0.8	7.9 ± 0.7	0.55
36 weeks (cm)	8.8 ± 0.8	8.7 ± 1.0	0.67

Data represents mean ± standard deviation or value (percent).

*cm* centimeter, *d* day, *EGA* estimated gestational age, *g* grams, *kg* kilogram, *yrs* years.

^a^Data represents median (interquartile range).

**Table 2:** Weight growth velocity at 28 days and 36 weeks postmenstrual age.

Outcome	Variable	Level of variable	Adjusted least squares mean (SE)	*F*-value	*p*-value
Weight velocity at day of life 28	Race			0.19	0.83
	Gender			1.28	0.26
	EGA at birth			3.07	0.09
	Birth weight			3.99	0.05
	Group	Early	13.6 (0.7)	0.00	1.00
		Late	13.6 (0.6)		
Weight velocity at 36 weeks	Race			0.72	0.49
	Gender			9.28	<0.01
	EGA at birth			4.40	0.04
	Birth weight			8.47	<0.01
	Group	Early	14.6 (0.6)	1.28	0.26
		Late	13.8 (0.6)		

Analysis of covariance controlling for race, gender, estimated gestational age, and birth weight.

**Fig. 2:**
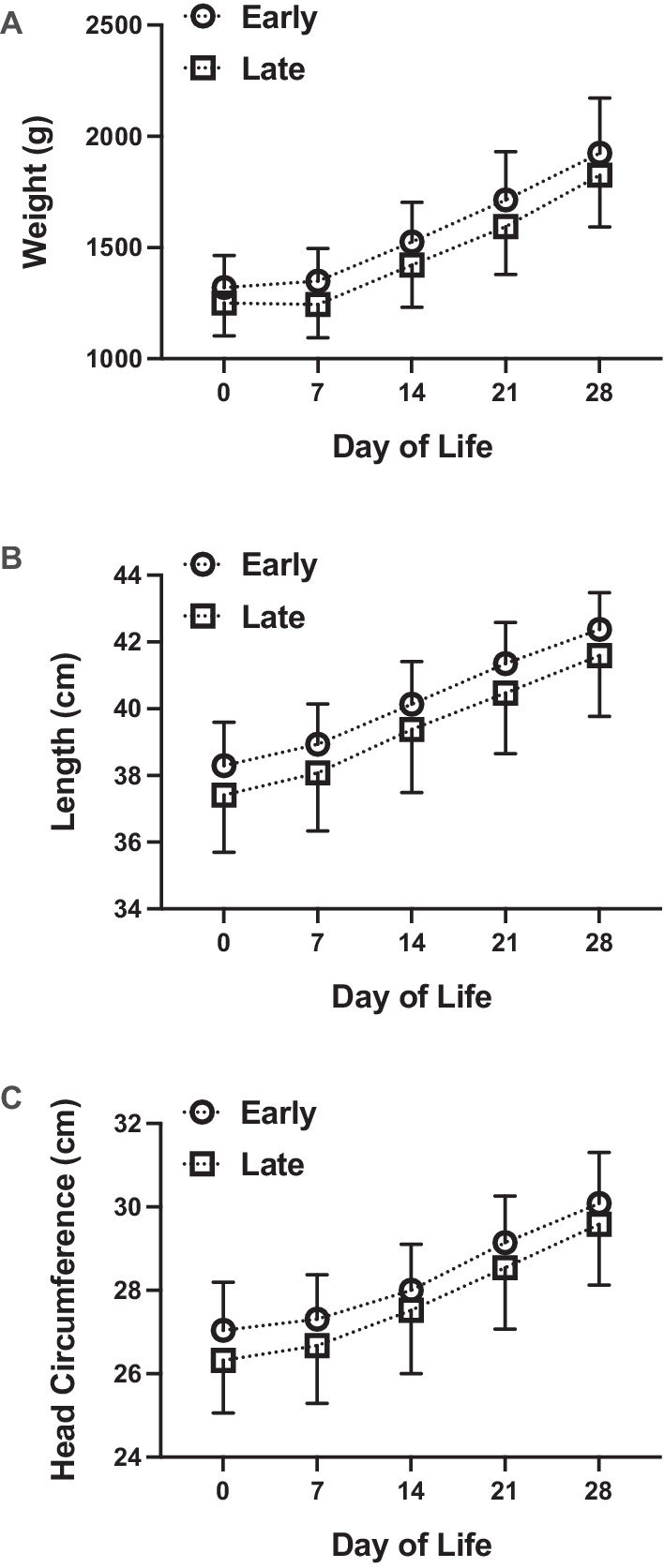
Weight, length, and head circumference over the first 4 weeks of life for study participants. Weight ((**A**), grams), length ((**B**), centimeters), and head circumference ((**C**), centimeters) over the first 28 days of life in very low birth weight infants randomly allocated to early (open circles) or late (open squares) fortification. Data represent mean ± standard deviation.

**Fig. 3:**
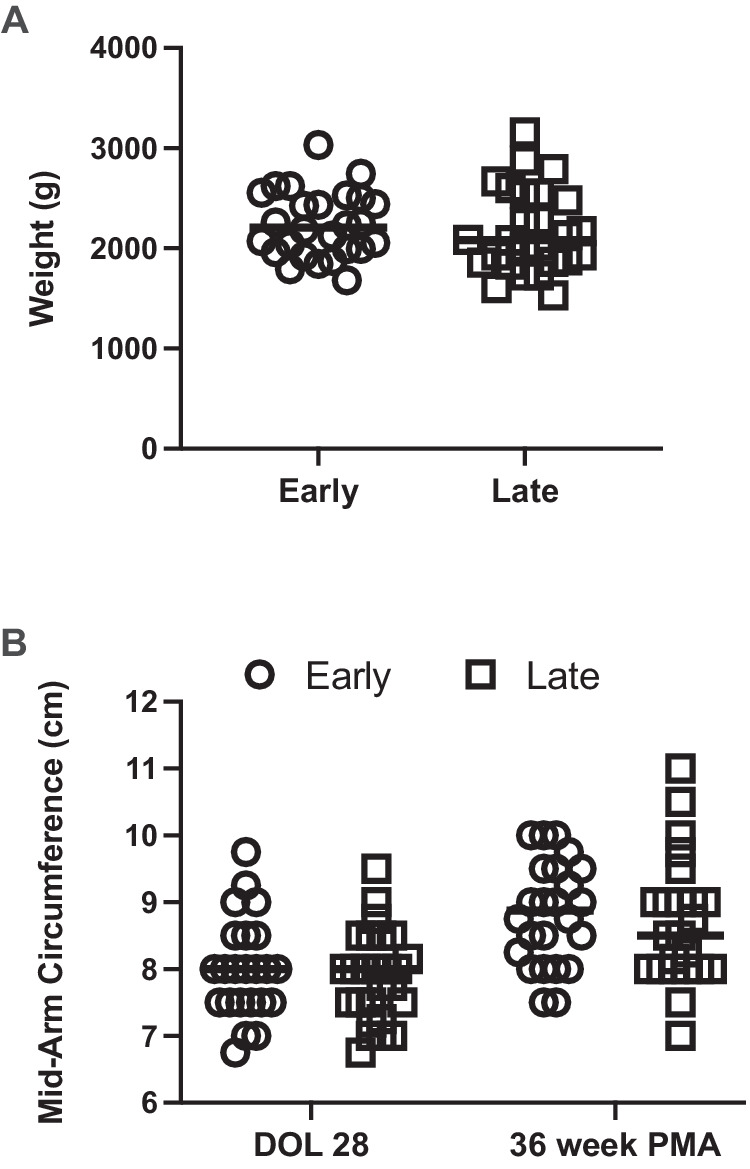
Weight and mid-arm circumference at 36 weeks postmenstrual age. **A** Weight (grams) at 36 weeks postmenstrual age in very low birth weight infants randomly allocated to early (open circles) or late (open squares) fortification. Bar represents mean for each group. **B** Mid-arm circumference (centimeters) at day of life (DOL) 28 and 36 weeks postmenstrual age in very low birth weight infants randomly allocated to early (open circles) or late (open squares) fortification. Bar represents mean for each group.

Several secondary outcomes were analyzed including weight, length, and head circumference velocity at 36 weeks PMA. No statistically significant difference between groups was observed for any growth velocity at 36 weeks PMA in univariate analysis (Table 1) or after controlling for race, gender, gestational age at birth, and birth weight (Table 2). We did observe that the Early fortification cohort tended to have higher weight, length, and head circumference measurements across the first 4 weeks of the study (Fig. 2A–C), but these relationships failed to reach statistical significance. Similarly, the weight at 36 weeks PMA did not differ statistically between groups (2237 ± 332 g vs. 2176 ± 417 g, *p* = 0.5648, Fig. 3A). We also measured mid-arm circumference at DOL 28 and 36 weeks PMA as an assessment of adiposity [19]. We did not identify a statistically significant difference between groups at DOL 28 or 36 weeks PMA (Table 1 and Fig. 3B).

We also assessed several feeding-related secondary outcomes. The bovine milk-derived HMF was added to human milk on DOL 1 in the Early group and DOL 9 in the Late group (*p* < 0.0001), but the total days of HMF or time to reach full feeds (i.e., ≥130 ml/kg/day) did not differ statistically between groups (Table 3). The total volume of human milk and ratio of mother’s own milk to donor human milk did not differ statistically between groups nor did the percentage of infants who were provided an exclusive donor human milk diet (Table 3). Similarly, no statistically significant difference in stool output, emesis volume or events, feeding-related NPO occurrence, or metabolic acidosis between groups was identified. Finally, the numbers of infants diagnosed with any NEC, oxygen at 36 weeks PMA, any retinopathy, or culture-positive late-onset sepsis did not differ between groups (Table 3).

**Table 3:** Outcomes by fortifier group.

	Early (*n* = 26)	Late (*n* = 26)	*p*-value
DOL HMF added	1 ± 0.6	9 ± 2.0	<0.01
Total HMF (d)	32 ± 14	31 ± 18	0.72
DOL full feeds (130 mL/kg/d)	12 ± 1.7	12 ± 2.0	0.51
Human milk			
Maternal (mL)	3495 ± 4596	4163 ± 6931	0.68
Donor (mL)	2411 ± 1892	2855 ± 2283	0.45
Maternal-to-donor ratio	0.39 ± 0.44	0.42 ± 0.44	0.81
Exclusive donor milk	13 (50)	11 (42)	0.58
HMF > 24 kcal/ounce	8 (31)	11 (42)	0.39
Total parenteral nutrition (d)	12 ± 6	11 ± 3	0.54
Stool (# per day)	2.6 ± 0.8	2.7 ± 0.87	0.78
Emesis (mL)	1.9 ± 2.5	1.5 ± 2.3	0.57
No emesis	14 (54)	16 (62)	0.57
Feeding related NPO occurrence^a^	7 (27)	6 (23)	0.75
Metabolic acidosis occurrence^a^	3 (12)	2 (8)	0.64
Diagnoses			
NEC			
Stage 1	0	0	–
Stage 2	1 (4)	0	1.0
Stage 3	0	0	–
Stage 4	0	0	–
Spontaneous intestinal perforation	0	0	–
Oxygen at 36 weeks PMA	5 (19)	3 (12)	0.44
Retinopathy (any stage)	0	0	–
Late-onset sepsis	0	0	–
Length of stay (d)	48 ± 21	50 ± 19	0.73

*d* day, *DOL* day of life, *HMF* human milk fortifier, *kcal* kilocalorie, *kg* kilogram, *mL* milliliter, *NEC* necrotizing enterocolitis, *NPO* “nothing by mouth”, *PMA* postmenstrual age.

Data represents mean ± standard deviation or value (percent).

^a^Occurrence may indicate > 1 episode in a single patient.

## Discussion

In this double-blind randomized controlled trial, we compared early growth-related outcomes in a random sample of VLBW infants provided a bovine milk-derived HMF on feeding day 1 with comparison to a similar cohort of VLBW infants provided a bovine milk-derived HMF after reaching ≥ 80 ml/kg/day of enteral feeding volume. The study successfully recruited 91% (52 of 57) of eligible infants during the study period, masked providers and parents to study allocation, retained 100% of enrolled participants throughout the study period, and successfully monitored anthropometrics and secondary outcomes throughout the study period for all infants. Our analysis indicated that addition of a bovine milk-derived HMF did not associate with higher weight velocity at DOL 28 nor did we identify any growth advantages in the intervention group at DOL 28 or 36 weeks PMA. We also did not identify any statistically significant differences between groups with regard to several secondary outcomes. To our knowledge, this is the first double-blind randomized clinical trial of early fortification using a bovine milk-derived HMF beginning with the first enteral feedings in VLBW and/or preterm infants.

The addition of HMFs to human milk is necessary for preterm infant growth. Human milk, regardless of its source, lacks sufficient quantities of protein, sodium, key minerals, and trace elements to support postnatal growth and development [5–7]. Despite widespread use of HMFs for preterm infants, the timing of fortification remains controversial due to a lack of high-quality studies. An emerging body of literature suggests that addition of HMFs prior to 80 or 100 ml/kg/day is safe and may provide some benefit to early or later growth outcomes. Salas and colleagues recently published a double-masked randomized clinical trial of 150 extremely preterm infants (<28 weeks PMA) provided a human milk-derived HMF on feeding day 2 and compared outcomes with “routine” fortification using a bovine milk-derived HMF on feeding day 14 [13]. No differences in body mass composition were identified (primary outcome) but they did identify some modest growth benefits including reduced early weight loss and improved length gains at term-corrected age. Importantly, the incidence of NEC, SIP, and death did not differ between groups. Conversely, Sullivan and colleagues demonstrated that addition of a human milk-derived HMF at a low enteral feeding volume (i.e., 40 ml/kg/day) did not benefit growth outcomes at term-corrected age but did appear to be well-tolerated. The authors did identify a higher incidence of NEC in a third arm of the study that compared early and delayed addition of a human milk-derived HMF to preterm infants who were randomly assigned to a bovine milk-derived HMF added at 100 ml/kg/day. However, this multicenter study was not masked and may introduce some diagnostic bias. Recent publication of a large open-label multicenter study (*n* = 229, <28 weeks PMA) demonstrated no difference in NEC, death, or sepsis for preterm infants provided a bovine milk-derived HMF when compared with those provided a human milk-derived HMF [20]. Relevant to the timing of fortification, both groups were exposed to HMF within the first week of life.

Early addition of human milk-derived HMFs is gaining acceptance as evidenced by published feeding protocols provided by one commercial entity and recent clinical reports [21–23]. Early addition of bovine milk-derived HMFs is more controversial with few studies to guide clinical decision-making. Shah et al. randomized 100 VLBW infants to open-label addition of a bovine milk-derived HMF at 20 ml/kg/day with comparison to fortification occurring at 100 ml/kg/day [16]. The number of days to full enteral feedings (primary outcome) did not differ between groups, occurring on postnatal day 20, and feeding-related morbidities were similar between groups. Infants randomized to early fortification experienced improved weight velocity at 28 days when compared to delayed fortification; however, the time to reach “full enteral feeds” in both groups was considerable. An earlier clinical trial from Iran randomized preterm (28–34 weeks PMA) and low birth weight (<2 kg) infants to receive a powder bovine milk-derived HMF beginning on feeding day 1 as compared to later fortification at 75 ml/kg/day demonstrating no significant growth benefits at 4 weeks with early fortification using a bovine milk-derived HMF [17].

Our study significantly advances these findings on several fronts. Recruitment and retention of eligible infants was high and both clinicians, parents, and investigators were blinded to study allocation until all infants were discharged from the hospital. Although early addition of a bovine milk-derived HMF did not appear beneficial to any growth outcomes at either DOL 28 or 36 weeks PMA, early fortification appears to be well-tolerated with no appreciable adverse outcomes. Infants in both arms reached “full enteral feeds” at DOL 12 and days of TPN did not differ between groups. Importantly, we tracked feeding-related outcomes in real time using both frequency and volume of emesis, stool count, and feeding-related NPO occurrence as noted by the clinical care team which was blinded to study allocation. Similarly, adverse outcomes did not differ between groups. It is likely that any potential growth benefits of early fortification were impaired by the conservative study design. First, we focused on a preterm population with relatively low risk for NEC and other adverse outcomes. Second, the early fortification strategy resulted in the bovine milk-derived HMF accounting for less than 10% (vol/vol) of the total enteral feeding volume during the intervention period. The minimal additional calories and nutrients provided by such a strategy and over a short period of time appear to exert minimal or no impact on growth.

In conclusion, we provide valuable clinical data that addition of a bovine milk-derived HMF is safe and well-tolerated in VLBW infants despite no clear growth benefits noted. Since bovine milk-derived HMFs are used by nearly 3 in 4 NICUs in the United States [10] and are commonly employed throughout the world, our findings should encourage clinicians to initiate fortification earlier in their feeding protocols and provide good rationale for future clinical trials. However, we also acknowledge that the gestational age and birth weight of the population under study was conservative and the safety or tolerability of immediate fortification of human milk with a bovine-based HMF in more immature infants must be examined in future prospective clinical trials.

## Data Availability

Data will be made available upon reasonable written request to the corresponding author.

## References

[1] Committee On N, Section On B, Committee On F, Newborn. Donor human milk for the high-risk infant: preparation, safety, and usage options in the United States. Pediatrics. 2017;139:e20163440. 27994111 10.1542/peds.2016-3440

[2] Agostoni C, Buonocore G, Carnielli VP, De Curtis M, Darmaun D, Decsi T, et al. Enteral nutrient supply for preterm infants: commentary from the European Society of paediatric gastroenterology, hepatology and nutrition committee on nutrition. J Pediatr Gastroenterol Nutr. 2010;50:85–91. 19881390 10.1097/MPG.0b013e3181adaee0

[3] Embleton ND, Moltu SJ, Lapillonne A, van den Akker CHP, Carnielli V, Fusch C, et al. Enteral nutrition in preterm infants (2022): a position paper from the ESPGHAN Committee On Nutrition And Invited Experts. J Pediatr Gastroenterol Nutr. 2023;76:248–68. 36705703 10.1097/MPG.0000000000003642

[4] Boundy EO, Anstey EH, Nelson JM. Donor human milk use in advanced neonatal care units—United States, 2020. MMWR Morb Mortal Wkly Rep. 2022;71:1037–41. 35980851 10.15585/mmwr.mm7133a1 PMC9400533

[5] Gates A, Hair AB, Salas AA, Thompson AB, Stansfield BK. Nutrient composition of donor human milk and comparisons to preterm human milk. J Nutr. 2023;153:2622–30. 37517552 10.1016/j.tjnut.2023.07.012

[6] Gates A, Marin T, De Leo G, Waller JL, Stansfield BK. Nutrient composition of preterm mother’s milk and factors that influence nutrient content. Am J Clin Nutr. 2021;114:1719–28. 34293087 10.1093/ajcn/nqab226 PMC10157816

[7] Gates A, Marin T, Leo G, Stansfield BK. Review of preterm human-milk nutrient composition. Nutr Clin Pr. 2020;36:1163–72. 10.1002/ncp.10570 PMC10037211 32862494

[8] Arslanoglu S, Boquien CY, King C, Lamireau D, Tonetto P, Barnett D, et al. Fortification of human milk for preterm infants: update and recommendations of the European Milk Bank Association (EMBA) Working Group on Human Milk Fortification. Front Pediatr. 2019;7:76. 30968003 10.3389/fped.2019.00076 PMC6439523

[9] Hair AB, Scottoline B, Good M. Dilemmas in human milk fortification. J Perinatol. 2023;43:103–7. 10.1038/s41372-022-01502-6 PMC10317058 36097287

[10] Perrin MT. Donor human milk and fortifier use in United States level 2, 3, and 4 neonatal care hospitals. J Pediatr Gastroenterol Nutr. 2018;66:664–9. 29045350 10.1097/MPG.0000000000001790

[11] Hilditch C, Keir A, Collins CT, Middleton P, Gomersall J. Early versus delayed introduction of human milk fortification in enterally fed preterm infants: a systematic review and meta-analysis. J Paediatr Child Health. 2022;58:30–38. 34669996 10.1111/jpc.15810

[12] Thanigainathan S, Abiramalatha T. Early fortification of human milk versus late fortification to promote growth in preterm infants. Cochrane Database Syst Rev. 2020;7:CD013392. 32726863 10.1002/14651858.CD013392.pub2 PMC7390609

[13] Salas AA, Gunawan E, Nguyen K, Reeves A, Argent V, Finck A, et al. Early human milk fortification in infants born extremely preterm: a randomized trial. Pediatrics. 2023;152:e2023061603. 37551512 10.1542/peds.2023-061603 PMC10471508

[14] Sullivan S, Schanler RJ, Kim JH, Patel AL, Trawoger R, Kiechl-Kohlendorfer U, et al. An exclusively human milk-based diet is associated with a lower rate of necrotizing enterocolitis than a diet of human milk and bovine milk-based products. J Pediatr. 2010;156:562–7.e561 20036378 10.1016/j.jpeds.2009.10.040

[15] Huston R, Lee M, Rider E, Stawarz M, Hedstrom D, Pence M, et al. Early fortification of enteral feedings for infants <1250 grams birth weight receiving a human milk diet including human milk based fortifier. J Neonatal Perinat Med. 2020;13:215–21. 10.3233/NPM-190300 PMC7369034 31707377

[16] Shah SD, Dereddy N, Jones TL, Dhanireddy R, Talati AJ. Early versus delayed human milk fortification in very low birth weight infants—a randomized controlled trial. J Pediatr. 2016;174:126–31.e121 27112041 10.1016/j.jpeds.2016.03.056

[17] Alizadeh Taheri P, Sajjadian N, Asgharyan Fargi M, Shariat M. Is early breast milk fortification more effective in preterm infants?: a clinical trial. J Perinat Med. 2017;45:953–7. 27676603 10.1515/jpm-2015-0375

[18] Walsh MC, Kliegman RM. Necrotizing enterocolitis: treatment based on staging criteria. Pediatr Clin North Am. 1986;33:179–201. 3081865 10.1016/S0031-3955(16)34975-6 PMC7131118

[19] Daly-Wolfe KM, Jordan KC, Slater H, Beachy JC, Moyer-Mileur LJ. Mid-arm circumference is a reliable method to estimate adiposity in preterm and term infants. Pediatr Res. 2015;78:336–41. 26020147 10.1038/pr.2015.103

[20] Jensen GB, Domellöf M, Ahlsson F, Elfvin A, Navér L, Abrahamsson T. Effect of human milk-based fortification in extremely preterm infants fed exclusively with breast milk: a randomised controlled trial. eClinicalMedicine. 2024;68:102375. 38545091 10.1016/j.eclinm.2023.102375 PMC10965410

[21] Fleig L, Hagan J, Lee ML, Abrams SA, Hawthorne KM, Hair AB. Growth outcomes of small for gestational age preterm infants before and after implementation of an exclusive human milk-based diet. J Perinatol. 2021;41:1859–64. 34012050 10.1038/s41372-021-01082-x PMC8342303

[22] Hair AB, Hawthorne KM, Chetta KE, Abrams SA. Human milk feeding supports adequate growth in infants </= 1250 grams birth weight. BMC Res Notes. 2013;6:459. 24220185 10.1186/1756-0500-6-459 PMC3879715

[23] Swanson JR, Becker A, Fox J, Horgan M, Moores R, Pardalos J, et al. Implementing an exclusive human milk diet for preterm infants: real-world experience in diverse NICUs. BMC Pediatr. 2023;23:237. 37173652 10.1186/s12887-023-04047-5 PMC10176849

